# Unusual spread of the monkeypox virus: An emerging threat to the public health and the possible containment

**DOI:** 10.1016/j.amsu.2022.104580

**Published:** 2022-09-07

**Authors:** Fahadul Islam, Manish Dhawan, Talha Bin Emran

**Affiliations:** Department of Pharmacy, Faculty of Allied Health Sciences, Daffodil International University, Dhaka, 1207, Bangladesh; Department of Microbiology, Punjab Agricultural University, Ludhiana, 141004, Punjab, India; Trafford College, Altrincham, Manchester, WA14 5PQ, UK; Department of Pharmacy, Faculty of Allied Health Sciences, Daffodil International University, Dhaka, 1207, Bangladesh; Department of Pharmacy, BGC Trust University Bangladesh, Chittagong, 4381, Bangladesh

**Keywords:** Monkeypox virus, Public health, Risk factor, Containment, Smallpox

Dear Editor,

The DNA virus known as the monkeypox virus induces monkeypox in both humans and other animals [1]. It is a member of the family Poxviridae and the genus Orthopoxvirus. In addition to the variola (VARV), cowpox (CPX), and vaccinia (VACV) viruses, it is a human orthopoxvirus. It is not a predecessor or a descendent of the variola virus, which causes smallpox. Monkeypox and smallpox are similar, although smallpox has a gentler rash and a lesser fatality rate [2]. The virus has two separate clades in the two places, called Congo Basin (Central African) and West African clades, respectively [1]. The virus's aggressiveness has varied in Central African isolates, with more virulent strains than those from Western Africa. The virus is primarily prevalent in Central and West African tropical forests. It was initially identified in monkeys in 1958, and it was discovered in humans in 1970. Over 400 human cases were documented between 1970 and 1986. Small viral epidemics with a mortality rate of 10% and a secondary human-to-human incidence rate of roughly the same percentage are frequent in tropical Central and West Africa. The most common way to become contaminated is thought to be through contact with an infected person or their body fluid. In 2003, the first outbreak outside of Africa was recorded, and there were no fatalities [3].

Primates and other animals can spread monkeypox virus as they have been found as the incidental hosts of the etiological agent. In 1958, Preben von Magnus found it in *Macaca fascicularis* that were utilized as study animals. The 2003 epidemic in the US was attributed to prairie dogs that were infected by a Gambian pouched rat that was exported from Ghana [1]. The monkeypox virus infects both primates and non-primate animals. The virus is primarily identified in Central and West African tropical rainforests [3]. Both human to human and animal to human transmission of the virus is conceivable. Animals can infect people by bites or direct interaction with bodily fluids of pathogens. The virus can spread between people through droplet inhalation and exposure with fomites (tactile objects) contaminated by an affected person's body fluid. The incubation phase lasts anywhere from 10 to 14 days. Before the rash appears, prodromal symptoms include lymph node swelling, muscle pain, headache, and fever [4].

The primary difference between the manifestations of smallpox and monkeypox is that smallpox does not result in swollen lymph nodes (lymphadenopathy), but monkeypox does. Monkeypox typically takes 7–14 days to incubate (from inoculation to manifestations), but it can take as little as 521 days. Temperature increased, headaches, neck discomfort, back pain, swollen lymph nodes, chills, and exhaustion are the first symptoms of the illness [5]. After the commencement of fever, the patient experiences a rash for 1–3 days (rarely longer), which typically begins on the face and extends to other parts of the body. Sores undergo the subsequent steps before disappearing: macules, pimples, vesicles, blisters, and scar tissue. On typically, the illness lasts for roughly 24 weeks. In Africa, monkeypox has been found to be fatal to up to one out of every ten individuals who contract it [6].

Avoid contacting with any animals that might be carrying the virus (includes animals that are ill or have been confirmed dead in monkeypox-prone regions). Isolate infected individuals from people who could contract the infection by not handling bedding or other items that have come into direct contact with a sick animal. Hands should be washed properly after coming into contact with sick animals or people. Two options include using an alcohol-based hand sanitizer or cleaning hands with warm water and soap. Use personal protective equipment (PPE) when taking care of patients. So, imagine washing your hands. After coming into contact with infectious animals or humans, wash your hands thoroughly [6]. JYNNEOSTM is an attenuation live virus vaccination, commonly known as Imvamune or Imvanex that has been authorized for the prevention of monkeypox by the US Food and Drug Administration. The Advisory Committee on Immunization Practices (ACIP) agreed on November 3, 2021, to recommend JYNNEOS pre-exposure prophylaxis as an alternative to ACAM2000 for some people at risk of orthopoxvirus infection [6].

It contains any possible outbreaks, making monitoring and early diagnosis identification vital actions that should be considered seriously. Transmission with sick people is the major manifestations for monkeypox virus infection during human monkeypox epidemics. Infected health personnel and household members are at a higher risk. Health professionals managing samples from individuals with a suspicion or confirmed monkeypox infectious agent should adhere to standard infection control practices. If possible, people who have been vaccinated against smallpox should be chosen to care for the patient. Clusters of monkeypox cases were discovered in numerous non-endemic nations in May 2022, with no direct travel links to an endemic location. More research is being conducted to find out the likely source of infection and to prevent further spread. While the origins of this outbreak are being explored, it's critical to consider all possible mechanisms of transmission to protect public health. The handling of samples obtained from individuals and animals suspicious of containing the monkeypox virus should take place in facilities with the necessary expertise. In compliance with WHO guidelines for transporting infectious substances, patient samples must be securely packaged three times before shipping [7,8].

## Ethical approval

Not applicable.

## Sources of funding

This study received no specific grant from any funding agency in the public, commercial, or not-for-profit sectors.

## Author contribution

Fahadul Islam: Conceptualization, Data Curation, Writing - Original Draft, Writing - review & editing. Manish Dhawan: Writing - Original Draft, Writing - review; editing. Talha Bin Emran: Supervision, Writing - Original Draft, Writing - review; editing. All authors critically reviewed and approved the final version of the manuscript.

## Registration of research studies

1. Name of the registry: Not applicable.

2. Unique Identifying number or registration ID.

3. Hyperlink to your specific registration (must be publicly accessible and will be checked): Not applicable.

## Consent

Not applicable.

## Guarantor

Talha Bin Emran, Ph.D., Associate Professor, Department of Pharmacy, BGC Trust University Bangladesh, Chittagong 4381, Bangladesh. T: +88-030-3356193, Fax: +88-031-2550224, Cell: +88-01819-942214. https://orcid.org/0000-0003-3188-2272. E-mail: talhabmb@bgctub.ac.bd.

## Data statement

No specific data collected for the above manuscript.

## Provenance and peer review

Not commissioned, internally peer-reviewed.

## Declaration of competing interest

All authors report no conflicts of interest relevant to this article.
